# Aldehyde Dehydrogenase 2 Mediates Alcohol‐Induced Colorectal Cancer Immune Escape through Stabilizing PD‐L1 Expression

**DOI:** 10.1002/advs.202003404

**Published:** 2021-03-24

**Authors:** Hong Zhang, Yuhui Xia, Fang Wang, Min Luo, Ke Yang, Shaobo Liang, Sainan An, Shaocong Wu, Chuan Yang, Da Chen, Meng Xu, Muyan Cai, Kenneth K. W. To, Liwu Fu

**Affiliations:** ^1^ Sun Yat‐sen University Cancer Center State Key Laboratory of Oncology in South China Collaborative Innovation Center for Cancer Medicine Guangdong Esophageal Cancer Institute Guangzhou 510060 China; ^2^ School of Pharmacy Faculty of Medicine The Chinese University of Hong Kong Hong Kong China

**Keywords:** alcohol, ALDH2, colorectal cancer, immune escape, PD‐L1

## Abstract

Despite the great success of immunotherapy in a small subset of cancer patients, most colorectal cancer (CRC) patients do not respond to programmed cell death receptor 1 (PD‐1) blockade immunotherapy. There is an urgent medical need to elucidate how cancer cells evade immune response and to develop novel means to boost the efficacy of immune checkpoint inhibitors. In this study, alcohol induces ligand programmed cell death receptor 1 (PD‐L1) expression of CRC cells in vitro and in vivo. Alcohol exposure is shown to induce aldehyde dehydrogenase 2 (ALDH2) expression that is a crucial enzyme involved in alcohol metabolism, and low level of lymphocytes infiltration in the murine CRC model and patients. Intriguingly, ALDH2 and PD‐L1 protein expression are positively correlated in tumor tissues from the CRC patients. Mechanistically, ALDH2 stabilizes PD‐L1 protein expression by physically interacting with the intracellular segment of PD‐L1 and inhibiting its proteasome‐dependent degradation mediated by an E3 ubiquitin ligase Speckle Type POZ Protein (SPOP). Importantly, inhibition of ALDH2 reduces PD‐L1 protein in CRC cells and promotes tumor‐infiltrating T cells (TILs) infiltration, presumably leading to the significant potentiation of anti‐PD‐1 antibody efficacy in a mouse CT26 CRC model. The findings highlight a crucial role played by ALDH2 to facilitate alcohol‐mediated tumor escape from immunity surveillance and promote tumor progression.

## Graphical abstract

Aldehyde dehydrogenase 2 (ALDH2) promotes alcohol‐mediated immune escape through stabilizing ligand programmed cell death receptor 1 (PD‐L1) protein expression by interacting with the intracellular segment of PD‐L1, thereby inhibiting its ubiquitination mediated by Speckle Type POZ Protein (SPOP). Silencing of ALDH2 significantly sensitizes tumors to PD‐1 blockade therapy.

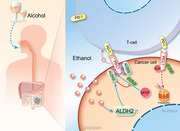

## 1 Introduction

Epidemiological evidence indicates that alcohol abuse is one of the most important risk factors for colorectal cancer (CRC).^[^ ^1^ ^]^ Excessive alcohol consumption is associated with immunosuppression, which abolishes the immune surveillance against tumor formation. Alcohol has been reported to impair the function and activation of T cells, and to induce T cell apoptosis.^[^ ^2^, ^3^, ^4^ ^]^ However, the precise mechanisms by which alcohol mediates T cell dysfunction to promote immune escape of CRC are unclear.

Programmed cell death receptor 1 (PD‐1) is a key inhibitory receptor residing on the surface of tumor‐infiltrating lymphocytes (TILs), when bound to its major ligand PD‐L1 expressing in tumor cells, will promote T cell exhaustion and blunt the immune response. The anti‐PD‐1/anti‐PD‐L1 immune checkpoint blockade therapy disrupts the PD‐1/PD‐L1 interaction and reactivates the anti‐tumor T‐cell‐mediated cell cytotoxicity by binding to the inhibitory PD‐1 receptor on TILs and PD‐L1 on tumor cells, respectively. In recent years, PD‐1/PD‐L1 immune checkpoint inhibitors have been approved by the US Food and Drug Administration for the treatment of CRC patients.^[^ ^5^ ^]^ Despite the great success of PD‐1/PD‐L1 blockade immunotherapy in a small subset of cancer patients, many patients do not respond and others eventually relapse due to adaptive resistance. To this end, overexpression of PD‐L1 in CRC is associated with immune escape and poor prognosis.^[^ ^6^ ^]^ To unravel the full potential of anti‐PD immunotherapy, intensive research has been conducted to elucidate the mechanisms underlying these de novo and adaptive resistance and to identify new strategies for their circumvention. Recently, cetuximab (an anti‐estimated glomerular filtration rate (EGFR) antibody) has been reported to enhance therapeutic effect of immunotherapy by inducing an EGFR‐specific T‐cell response and PD‐L1 immune checkpoint expression in CRC.^[^ ^7^ ^]^ The combination of PD‐1 inhibitor with the FOLFOX regimen (5‐fluorouracil, folinic acid, and oxaliplatin) was also shown to enhance anticancer immune response in CRC by increasing expression of PD‐L1 and CD8^+^ T‐cell infiltration.^[^ ^8^ ^]^ However, little is known about the mechanism of PD‐L1 regulation in CRC.

It was reported that PD‐L1 expression in oral squamous cell carcinoma was associated with alcohol consumption ^[^ ^9^ ^]^, but the mechanism remains elusive. Aldehyde dehydrogenase 2 (ALDH2) is a key enzyme involved in alcohol metabolism and it also converts toxic acetaldehyde to acetate. Data from a recent study analyzing 116 melanoma tumors revealed that high ALDH2 expression was associated with favorable immunotherapy response.^[^ ^10^ ^]^ Interestingly, alcohol consumption has been reported to induce high expression of ALDH2 in tumor tissues.^[^ ^11^ ^]^ We hypothesized that ALDH2 may modulate immune escape induced by alcohol. In the present study, alcohol was shown to induce PD‐L1 expression in CRC cells. After alcohol exposure, CRC‐tumor‐bearing mice exhibited higher response to anti‐PD‐1 treatment. Alcohol consumption was also found to induce ALDH2 expression in mice and tumor tissues of CRC patients. Moreover, ALDH2 was found to promote PD‐L1 protein stability. Importantly, the combination of ALDH2 inhibition and PD‐1/PD‐L1 checkpoint inhibitor was shown to enhance the anti‐tumor activity of effector immune cells. In summary, our findings revealed that alcohol consumption could induce ALDH2 and subsequently upregulate PD‐L1 expression in CRC to allow their escape from immune surveillance. Another salient point from the study is that inhibition of ALDH2 combined with anti‐PD‐1 treatment could be used as a novel strategy to potentiate immune blockade therapy in CRC patients especially in the alcoholic population.

## 2 Results

### 2.1 Alcohol Upregulated PD‐L1 Expression in CRC In Vitro and In Vivo

**Figure 1:**
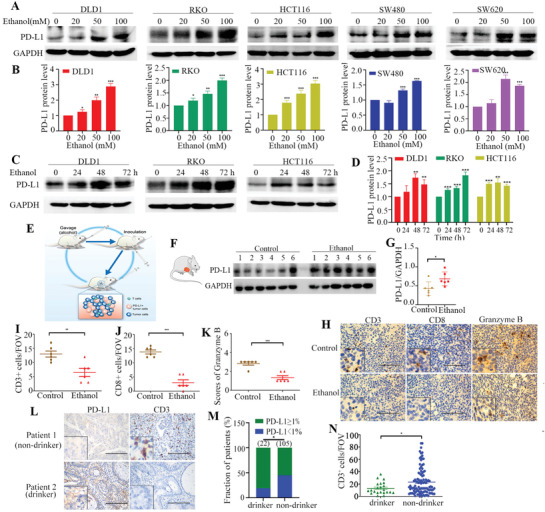
Alcohol induced PD‐L1 expression in vitro and in vivo. A) PD‐L1 protein expression was measured by western blot in five human colon cancer cell lines (DLD1, RKO, HCT116, SW480, and SW620) after treatment with ethanol at different concentrations (0 × 10^−3^, 20 × 10^−3^, 50 × 10^−3^, or 100 × 10^−3^ m) for 48 h. B) Relative PD‐L1 protein levels (normalized with GAPDH) in cells after treatment with different concentrations of ethanol for 48 h are presented as mean ±SD in bar graph. C) PD‐L1 protein expression in cells treated with 100 × 10^−3^ m ethanol for indicated time points. D) Relative PD‐L1 protein levels (normalized with GAPDH) in cells after treatment with 100 × 10^−3^ m ethanol for different time points are presented as mean ± SD in bar graph. E) Schematic diagram describing the mouse CT26 tumor model for investigating alcohol‐induced ALDH2 expression. F) Western blot analysis of PD‐L1 protein expression in tumor tissues from mice treated with control and ethanol (*n* = 6 in each animal group). G) Relative PD‐L1 protein levels (normalized with GAPDH) in tumor tissues from control or ethanol‐treated mice are presented in scatter plot (*n* = 6 in each animal group). H) Representative IHC staining images of CD3, CD8, and granzyme B from control or ethanol‐treated BALB/c mice. I,J) Quantification of CD3‐ and CD8‐positive cells are quantified per FOV in tumor tissues (400×). K) The immunoreactivity score of granzyme B in tumor tissues (400×). L) Representative IHC staining images of CD3 and PD‐L1 in tumor tissues from patient 1 (non‐drinker) and patient 2 (drinker). M) There is a significant association between alcohol intake and PD‐L1 expression in CRC patients (data were analyzed by chi‐square test). N) CD3 staining positive cells are quantified per FOV. Scale bar = 100 µm. Error bars represent SD. (Unpaired two‐tailed Student's *t*‐test, *p < 0.05; **p < 0.01; ***p < 0.001.) Experiments in A and C were all repeated for three times independently with similar results.

It is known that chronic and heavy drinking is closely linked to immunosuppression.^[^ ^12^ ^]^ A panel of CRC cell lines was treated with different concentration of ethanol in vitro. Ethanol was found to significantly upregulate PD‐L1 protein expression in a concentration and time dependent manner (**Figure** **1**A–D). However, the quantitative polymerase chain reaction (qPCR) results suggested that ethanol did not affect PD‐L1 expression at the mRNA level (Figure S1A, Supporting Information). The possible association between alcohol consumption and PD‐L1 expression in CRC tumor in animal model was then investigated by using immunohistochemical and western blot analysis. BALB/c mice were administered with ethanol (5 g kg^–1^, once daily) by gavage for 2 weeks before CT26 cells injection. Ethanol administration was continued for about another 3 weeks after tumor injection. Significantly higher PD‐L1 protein expression was detected in tumor tissues from mice receiving ethanol treatment than those without (Figure 1E,F). Moreover, immunohistochemistry (IHC) analysis indicated that biomarkers for T‐cell activation (including CD3, CD8, and granzyme B) detected on tumor cells of mice exposed to ethanol were remarkably lower than those in control mice (Figure 1G–J). Furthermore, the protein expression of PD‐L1 in tumor tissues of 127 CRC patients with or without alcohol drinking history was examined by IHC staining. Tumor tissues of CRC patients with alcohol drinking history were found to express significantly higher level of PD‐L1 and but lower CD3 than those from the non‐drinkers (Figure 1K–M). Hence, alcohol consumption was shown to promote CRC immune escape by upregulating PD‐L1 but reducing TILs in both mouse tumor model and CRC tumor specimens.

### 2.2 ALDH2 Induced PD‐L1 Expression in CRC In Vitro and In Vivo

**Figure 2:**
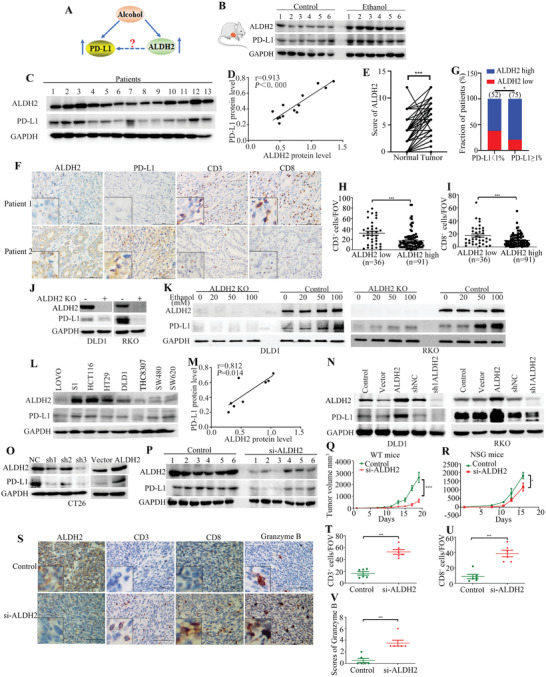
ALDH2 upregulated PD‐L1 expression in vitro and in vivo. A) The relationship among alcohol, PD‐L1, and ALDH2. B) Western blot analysis showing ALDH2 expression in tumor tissues from control and ethanol induced mice (*n* = 6 in each animal group). C) Protein expressions of ALDH2 and PD‐L1 from CRC patient tumor tissues were detected by western blot analysis (*n* = 13). D) Linear regression analysis plotting ALDH2 against PD‐L1 protein expression from 13 CRC patients (*r* = 0.913, *p* < 0.0001). E) ALDH2 expression scores in tumor and adjacent normal colon tissues. F) Expression of ALDH2, PD‐L1, CD3, and CD8 in tumor tissues of patients with ALDH2 low expression and high expression. G) The association between ALDH2 and PD‐L1 expression in tumor tissues from CRC patients. (Data were analyzed by chi‐square test.) H,I) Quantification of CD3‐ and CD8‐positive cells per FOV in tumor tissues (400×). J) Knockout ALDH2 decreases PD‐L1 expression in DLD1 and RKO cells. K) PD‐L1 protein expression was measured by western blot in DLD1 and RKO cells after treatment with ethanol at different concentrations (0 × 10^−3^, 20 × 10^−3^, 50 × 10^−3^, or 100 × 10^−3^ m) for 48 h. L) Western blot analysis of protein expressions of ALDH2 and PD‐L1 in eight human colon cancer cell lines. M) Linear regression analysis plotting ALDH2 against PD‐L1 protein expression from human colon cancer cell lines (*r* = 0.812, *p* = 0.014). N,O) Knockdown and overexpression of ALDH2 decreases and upregulates PD‐L1 expression in DLD1, RKO, and CT26 cells. P) Western blot analysis showing ALDH2 and PD‐L1 protein expressions in tumor tissues from control and si‐ALDH2‐treated mice. Q,R) The tumor volume analysis of control and si‐ALDH2 CT26 tumors in wild‐type (WT) and NSG mice. S) Representative IHC staining images of ALDH2, CD3, CD8, and granzyme B for BALB/c mice treated with control siRNA or si‐ALDH2 treatment. T,U) CD3 and CD8 staining positive cells were quantified per FOV. V) The immunoreactivity score of granzyme B in tumor tissues from control and si‐ALDH2 mice (400×). Scale bar = 100 µm. Error bars represent SD. (Unpaired two‐tailed Student's *t*‐test, *p < 0.05; **p < 0.01; ***p < 0.001.) All western blot analyses were repeated for three times independently with similar results.

ALDH2 is a major enzyme responsible for alcohol metabolism and it is known to be induced by alcohol consumption. We tested the underlying connection between alcohol and the PD‐L1 expression above (**Figure** **2**A). ALDH2 protein expression was found to be remarkably higher in tumor tissues collected from mice after ethanol administration than those without ethanol treatment (Figure 2B). This observed correlation between alcohol consumption and ALDH2 expression was further investigated in CRC patients. By IHC staining, 127 CRC specimens were ranked as displaying negative, low, median, and high ALDH2 expression (Figure S3A, Supporting Information). Alcohol drinkers were found to have significantly higher ALDH2 expression in their tumors than the non‐drinkers (**Table** **1**; Figure S3B, Supporting Information). To investigate the possible mechanistic relationship between alcohol consumption and CRC immune escape, protein expression levels of ALDH2 and PD‐L1 in tumor tissues from 13 CRC patients were evaluated (Figure 2C). Linear regression analysis indicated that ALDH2 was strongly and positively correlated with PD‐L1 protein expression in the CRC tumor specimens (*r* = 0.913, *p* < 0.0001) (Figure 2D). Also, ALDH2 expression was found to be higher in tumor tissues than the adjacent normal tissues (Figure 2E). Importantly, data from the IHC staining showed that CRC tissues exhibiting higher ALDH2 expression also had higher PD‐L1 level and vice versa (Figure 2F,G). Furthermore, CRC tissues displaying low ALDH2 expression were accompanied by more CD3^+^‐ and CD8^+^‐activated T cells infiltrated in the tumor, and vice versa (Figure 2F,H,I).

**Table 1:** Clinical characteristics of patients

Characteristics	Total (*n*)	ALDH2 low (*n*)	ALDH2 high (*n*)	*p*‐Value (*p*‐value by chi‐square test or Fisher's exact test (two‐sided test))
Gender				
Male	78	20	58	0.393
Female	49	16	33	
Age [years]				
<60	53	13	40	0.419
≥60	74	23	51	
TNM stage				
I+II	52	12	40	0.273
III+IV	75	24	51	
Differentiation degree				
High+middle	97	27	70	0.818
Low	30	9	21	
Alcohol				
No	105	34	71	0.036
Yes	22	2	20	

To confirm whether alcohol promoted PD‐L1 expression was through ALDH2, we performed western blot and found that ethanol significantly increased PD‐L1 protein level at the presence of ALDH2, while the upregulation of PD‐L1 expression was attenuated in ALDH2 knockout in RKO and DLD1 cells (Figure 2J,K). The correlation between ALDH2 and PD‐L1 protein expression in eight colon cancer cell lines was also examined by western blot analysis (Figure 2L). A strong and positive correlation between ALDH2 and PD‐L1 protein expression was also observed (*r* = 0.812, *p* = 0.014) (Figure 2M). Stable cell lines with ectopic overexpression or silencing of ALDH2 were established from DLD, RKO, and CT26 CRC cell lines. Forced expression of ALDH2 was found to significantly upregulate PD‐L1 expression, whereas knockdown of ALDH2 was shown to remarkably downregulate PD‐L1 in all stable cell line models (Figure 2N,O). Flow cytometry was used to detect the cell surface expression of PD‐L1. The increase and reduction of membranous PD‐L1 protein expression on cancer cells was caused by forced overexpression and knockdown of ALDH2, respectively (Figure S2C–F, Supporting Information). However, ALDH2 was showed to regulate PD‐L1 protein expression without affecting PD‐L1 mRNA level (Figure S1B, Supporting Information).

Next, ALDH2 inhibition was found to significantly downregulate PD‐L1 expression (Figure 2P; Figure S4A, Supporting Information). In addition, CT26 cells transfected with either a control siRNA or siRNA specifically against ALDH2 were implanted in wild type (immunocompetent) or immunodeficient NOD.Cg‐*Prkdc^scid^ Il2rg^tm1Wjl^/*SzJ (NSG) mice. While immunodeficient NSG mice implanted with si‐ALDH2‐transfected CT26 only exhibited a modest reduction in tumor volume (*p *< 0.05) compared with control CT26 cells (Figure 2Q), the immunocompetent mice (wild‐type) implanted with si‐ALDH2‐transfected CT26 cells displayed a significantly slower tumor growth (*p *< 0.01) than control CT26 (Figure 2R). The IHC staining results further showed that CD3+, CD8+, and granzyme B+ lymphocytes were significantly increased in the group of si‐ALDH2 (Figure 2S–V). These results indicate that ALDH2 may be the regulator for alcohol induced CRC immune escape.

### 2.3 ALDH2 Interacted with PD‐L1 Cytoplasmic Region

**Figure 3:**
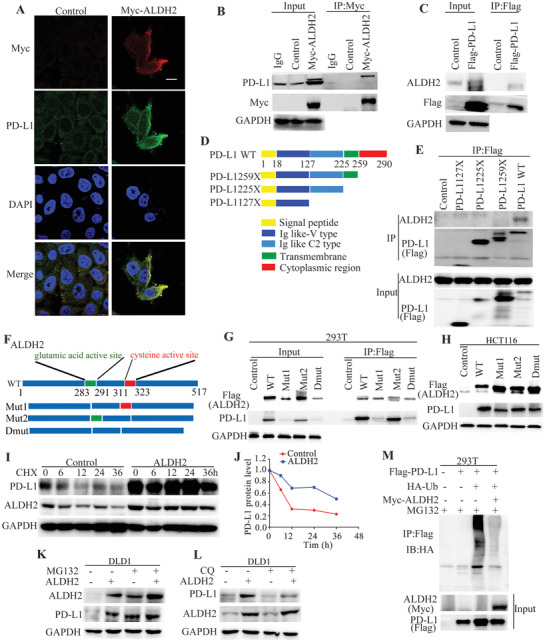
ALDH2 physically interacted with PD‐L1 through binding to its cytoplasmic domain. A) Immunofluorescence imaging of endogenous PD‐L1 and exogenous Myc‐ALDH2 proteins in THC8307 cells. Scale bars indicate 20 µm. B,C) The interaction between ALDH2 and PD‐L1 was confirmed by co‐IP assay. D) A map showing the three constructs of mutant PD‐L1 with different truncated domains. E) Data from Co‐IP assay revealed that ALDH2 interacts with the cytoplasmic region (amino acid residues 260–290) of PD‐L1. F) A schematic diagram showing the glutamic acid (283–291) and cysteine active site (311–323) of ALDH2. G) Co‐IP assay showing that the interaction between PD‐L1 and different ALDH2 mutants in 293T cells. H) Western blot detecting the effects of different ALDH2 mutants on PD‐L1 expression in HCT116 cells. I) Increased PD‐L1 protein stability was observed by CHX assay after ectopic overexpression of ALDH2 in DLD1 cells. J) *X*–*Y* graph comparing the rate of PD‐L1 protein degradation with or without ALDH2 overexpression. K,L) Western blot analysis investigating the effect of proteasome inhibitor (MG132, 30 × 10^−6^ m) or lysosomal inhibitor (chloroquine, 50 × 10^−6^ m) on ALDH2 and PD‐L1 protein expression in DLD1 cells with or without ALDH2 overexpression cells were harvested at 12 h after MG132 or chloroquine incubation. M) In vitro ubiquitin conjugation assay showing the increased ubquitination of PD‐L1 in the presence of ALDH2 ectopic overexpression. Whole cell lysates of 293T cells transfected with the indicated constructs were harvested at 12 h after incubation with MG132 (30 × 10^−6^ m). All western blot analyses were repeated for three times independently with similar results.

By immunofluorescence staining, myc‐tagged ALDH2 was shown to co‐localize with endogenously expressed PD‐L1 on cancer cell surface (**Figure** **3**A). The physical interaction between ALDH2 and PD‐L1 proteins was further confirmed by co‐immunoprecipitation (Co‐IP) using either myc‐tagged ALDH2 or Flag‐tagged PD‐L1 as the bait protein (Figure 3B,C). To investigate the binding domain of the two proteins, three constructs of mutant PD‐L1 with different truncated domains (Figure 3D) were prepared. Co‐IP was performed using ALDH2 and the wild type PD‐L1 or one of the three truncated PD‐L1 proteins. The data indicated that truncation of the cytoplasmic region of PD‐L1 (260–290) could effectively disrupt the interaction between PD‐L1 and ALDH2 (Figure 3E). A glutamic acid and a cysteine residue have been implicated in the catalytic activity of mammalian ALDH2 (https://prosite.expasy.org/). To better understand how ALDH2 bound PD‐L1, the glutamic acid (284–291) and cysteine residue active sites (312–323) of ALDH2 were deleted and the binding activity of PD‐L1 was tested respectively in vitro (Figure 3F). Co‐IP indicates that both Mut1 (glutamic acid active site) and Mut2 (cysteine residue active site) of ALDH2 are unnecessary for the binding of PD‐L1 (Figure 3G). The upregulation of PD‐L1 was also triggered by additional ALDH2 mutants in HCT116 cells (Figure 3H). These results suggest that the interaction between ALDH2 and PD‐L1 does not rely on the catalytic domain of ALDH2. It has been reported that the cytoplasmic region of PD‐L1 could be poly‐ubiquitinated and this post‐translational modification regulates PD‐L1 protein stability.^[^ ^13^, ^14^ ^]^ To better understand the stabilization of PD‐L1 protein by ALDH2, cycloheximide (CHX)‐chase assay was conducted to estimate the half‐life of PD‐L1 protein. In ALDH2‐overexpressing cells, the half‐life of PD‐L1 protein was significantly increased (Figure 3I,J). Moreover, proteasome inhibitor MG132 (30 × 10^−6^ m) was shown to increase the protein expression of both PD‐L1 and ALDH2 (Figure 3K), whereas the lysosomal inhibitor chloroquine (50 × 10^−6^ m) displayed no effect on expression of the two proteins (Figure 3L). Furthermore, by in vitro ubiquitin conjugation assay, ALDH2 was confirmed to stabilize PD‐L1 by its reducing ubiquitin‐proteasomal degradation (Figure 3M).

### 2.4 ALDH2 Inhibited Proteasome‐Dependent Degradation of PD‐L1

**Figure 4:**
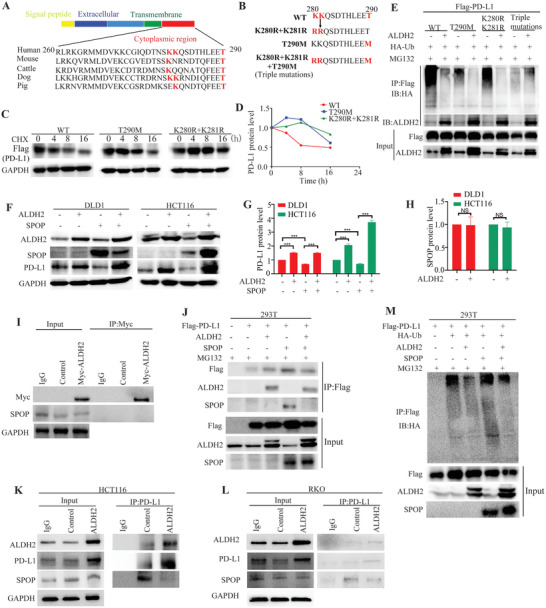
ALDH2 promoted proteasome‐dependent degradation of PD‐L1. A) Sequence alignment of the cytoplasmic region (260–290) of PD‐L1 in mammalian species. Amino acid residues at K280, K281, and T290 are conserved across different mammalian species. B) Additional mutants for mapping the ubiquitination sites of PD‐L1. C) CHX chase assay comparing the protein degradation rate of Flag‐tagged wild‐type PD‐L1 protein and two PD‐L1 mutants (T290M and K280R+K281R) in 293T cells. D) *X*–*Y* graph plotting the protein degradation rate of the Flag‐tagged PD‐L1 proteins obtained in (C). E) In vitro ubiquitin conjugation assay comparing the extent of ubquitination of wild‐type PD‐L1 and the three PD‐L1 mutants (T290M, K280R+K281R, and T290M+T280R+T281R) with or without ALDH2 overexpression in 293T cells. Whole cell lysates were harvested after incubation with MG132 (30 × 10^−6^ m) for 12 h. F) DLD1 and HCT116 cells are harvested for western blot analysis showing the effect of SPOP ectopic overexpression on ALDH2 and PD‐L1 expression in DLD1 and HCT116 cells. Whole cell lysates were harvested at 48 h after transfection. G) Relative PD‐L1 expression after normalization with GAPDH (mean ± SD) from (F) was plotted in bar graph. H) Relative ALDH2 expression after normalization with GAPDH (mean ± SD) from (F) was plotted in bar graph. I) Co‐IP assay demonstrating the interaction between ALDH2 and SPOP. J,M) Co‐IP assay showing the competition of ALDH2 with SPOP for binding to PD‐L1 and reduced ubquitination of PD‐L1 when ALDH2 competed with SPOP for binding to PD‐L1. Whole cell lysates of 293T were harvested at 12 h after incubation with MG132 (30 × 10^−6^ m). K,L) Co‐IP assay revealed that the competition of ALDH2 with SPOP for binding to PD‐L1 in HCT116 and RKO cells. All western blot analyses were repeated for three times independently with similar results.

Potential ubiquitination sites at the cytoplasmic region of PD‐L1 were predicted using UbPred (a computational tool for detection of post‐translational modification; http://www.ubpred.org/). The amino acid residues K280 and K281 of PD‐L1 were found to be the most likely ubiquitination sites. Recently, it has been reported that SPOP mediated poly‐ubiquitination and degradation of PD‐L1 protein by binding to the T290 site of PD‐L1.^[^ ^14^ ^]^ To this end, K280, K281, and T290 sites of PD‐L1 are highly relatively conserved among mammalian species (**Figure** **4**A). Three PD‐L1 expression constructs were prepared with a single mutation at T290M, double mutations at K280R+K281R, or triple mutations at K280R+K281R+T290M (Figure 4B). CHX chase assay was then conducted to evaluate the protein stability of PD‐L1 bearing these mutations. The results showed that the mutants of K280R+K281R and T290M could significantly prolong the protein half‐life of PD‐L1 (Figure 4C,D). Moreover, data from ubiquitination conjugation assay showed that the K280R+K281R and T290M mutants could significantly inhibited ubiquitination of PD‐L1 compared to the wild‐type PD‐L1 (Figure 4E). Furthermore, the concomitant PD‐L1 mutations of K280R+K281R+T290M was shown to retard ubiquitination and increase stability of the resulting protein, which was similar to the extra PD‐L1 stability achieved by co‐transfection of wild‐type PD‐L1 and ALDH2 (Figure 4E). SPOP, an E3 ubiquitinated ligase, was reported to facilitate ubiquitination of PD‐L1 at T290 site.^[^ ^14^ ^]^ Western blot analysis found that ALDH2 did not affect SPOP protein expression and could antagonize the inhibitory effect of SPOP on PD‐L1 (Figure 4F–H). Co‐IP experiment was performed to evaluate whether there was an interaction between ALDH2 and SPOP. The result showed that there was no interaction between ALDH2 and SPOP (Figure 4I). Instead, ALDH2 was shown to compete with SPOP to bind with PD‐L1 (Figure 4J–L), subsequently preventing the poly‐ubiquitination modification of PD‐L1 by SPOP (Figure 4M). Collectively, our results demonstrate that ALDH2 binds to the intracellular segment of PD‐L1 (260–290) and inhibits the ubiquitination modification at positions of K280, K281, and T290. ALDH2 was further shown to compete with SPOP for binding to PD‐L1, whereby poly‐ubiquitination modification of PD‐L1 by SPOP was inhibited.

### 2.5 Alcohol Consumption Enhanced Anticancer Effect from PD‐1 Blockade Treatment

**Figure 5:**
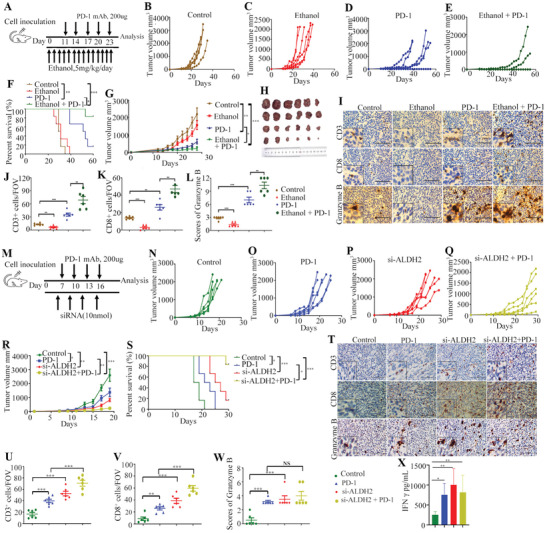
Combination therapy of alcohol exposure or ALDH2 knockdown and anti‐PD‐1 mAb in mouse CT26 colon cancer model. A) Schematic diagram illustrating the treatment plan for mice injected subcutaneously with CT26 cells. Female BALB/c mice are inoculated with 2 × 10^5^ CT26 cells subcutaneously and treated with or without anti‐PD‐1 mAb for five cycles. B–E) Tumor volume of mice treated with control (*n* = 6), ethanol (5 g kg^–1^ once daily, *n* = 6), anti‐PD‐1 mAb (200 µg, *n* = 6), and ethanol combined anti‐PD‐1 mAb therapy (*n* = 6) as indicated are measured every 2 days and plotted individually. F) Kaplan–Meier survival curves demonstrating the efficacy of different treatment groups on animal survival (log‐rank test). G) The tumor volume curves of five different groups (mean ± SD values were plotted). H) Photo showing individual tumors excised from different treatment groups at termination of study. I) Representative IHC staining results of CD3, CD8, and granzyme B from four treatment groups as indicated. J,K) The number of CD3‐ and CD8‐positive cells per FOV in tumor tissues from IHC analysis (400×). L) The immunoreactivity score of granzyme B in tumor tissues of different treatment groups (400×). M) Schematic diagram showing the treatment plan for mice injected subcutaneously with CT26 cells. Female BALB/c mice are inoculated with 3 × 10^5^ CT26 cells subcutaneously and treated with anti‐PD‐1 mAb or si‐ALDH2 for four cycles. N–Q) Tumor volume of mice treated with control (*n* = 6), anti‐PD‐1 mAb (*n* = 6), si‐ALDH2 (*n* = 6), and si‐ALDH2 combined anti‐PD‐1 mAb therapy (*n* = 6) as indicated were measured every 2 days and plotted individually. R) The tumor volume curves of four different groups. S) Kaplan–Meier survival curves showing the efficacy of different treatment groups (log‐rank test). T) Representative IHC staining results of CD3, CD8, and granzyme B from four treatment groups as indicated. U,V) The number of CD3‐ and CD8‐positive cells per FOV in tumor tissues from IHC analysis (400×). W) The immunoreactivity score of granzyme B of tumor tissues from different treatment groups (400×). X) ELISA analysis of the IFN‐*γ* level from serum samples of mice in different treatment groups. Scale bar = 100 µm. Error bars represent SD. (Unpaired two‐tailed Student's *t‐*test, ns = not significant, *p < 0.05; **p < 0.01; ***p < 0.001.)

A murine CT26 colon cancer model was employed to investigate the effect of alcohol consumption on PD‐1 blockade therapy (**Figure** **5**A). While alcohol consumption (5 g kg^–1^, once daily) did not affect tumor growth or animal survival in tumor‐bearing BALB/c mice, the addition administration of anti‐PD‐1 antibody to mice with alcohol consumption led to a more significant delay in tumor growth and prolonged survival compared to those receiving anti‐PD‐1 antibody alone without alcohol consumption (*p* < 0.05, and *p* < 0.05, respectively, Student's *t*‐test; Figure 5B–H). The IHC results further showed that CD3+, CD8+, and granzyme B+ cells were significantly increased in the group of combined alcohol consumption and anti‐PD‐1 antibody treatment compared to the mice with anti‐PD‐1 antibody treatment alone (*p* < 0.01, *p* < 0.01, and *p* < 0.01, respectively, Student's *t*‐test; Figure 5I–L).

### 2.6 ALDH2 Inhibition Enhanced Efficacy of PD‐1 Blockade in CT26 Tumor Model

Another murine CT26 colon cancer model was employed to investigate the effect of ALDH2 inhibition on PD‐1 immunotherapy (Figure 5M). The knockdown of ALDH2 by specific siRNA was found to potentiate the sensitivity of CT26 tumors to anti‐PD‐1 antibody, leading to a dramatically reduction in tumor burden (*p* < 0.01, Student's *t*‐test; Figure 5N–R; Figure S4B–G, Supporting Information), and improved overall survival compared with anti‐PD‐1 treatment alone (*p* < 0.05, Student's *t*‐test; Figure 5S). Interestingly, mice treated with ALDH2 siRNA alone also exhibited significantly retarded tumor progression (*p* < 0.01, Student's *t*‐test; Figure 5P,R,S; Figure S4F,G, Supporting Information), and improved overall survival (*p* < 0.001, Student's *t*‐test; Figure 5S), compared with the control group. The IHC results further showed that CD3+ and CD8+ T lymphocytes were significantly increased in the group of combined ALDH2 knockdown and anti‐PD‐1 antibody treatment (Figure 5T–V; Figure S4H–J, Supporting Information), and the expression of granzyme B was also moderately increased (Figure 5W; Figure S4K, Supporting Information). It is also noteworthy that the ALDH2 inhibition alone group exhibited increased TILs infiltration into the tumor tissues (Figure 5U–W). Furthermore, data from enzyme‐linked immunosorbent assay (ELISA) analysis showed that the plasma level of interferon gamma (IFN‐*γ*) in mice treated with si‐ALDH2 or anti‐PD‐1 antibody alone or si‐ALDH2 plus anti‐PD‐1 antibody treatment was all significantly higher than the control group (*p* < 0.05, *p* < 0.01, and *p* < 0.01, Student's *t*‐test; Figure 5X). Collectively, our findings indicated that ALDH2 reduction enhanced sensitivity of CT26 tumors to PD‐1 checkpoint blockade.

## 3 Discussion

CRC is a major cause of cancer‐related death worldwide. The occurrence of CRC is closely related to heredity, tobacco and alcohol intake, ulcerative colitis, viral infection, and environmental factors.^[^ ^15^ ^]^ Alcohol consumption is estimated to increase the risk of CRC by 60%. ^[^ ^16^ ^]^ Moreover, the association between alcohol intake and risk of CRC was known to be dose‐dependent. Heavy drinking is linked with a significantly increased risk of morbidity and mortality.^[^ ^17^, ^18^ ^]^ It has been proposed that alcohol inhibits T cell activation to suppress anti‐tumor immune response.^[^ ^19^, ^20^ ^]^ On the other hand, high expression of PD‐L1 is known to be associated with CRC immune evasion and poor prognosis.^[^ ^21^ ^]^ Here, we reported that alcohol induced PD‐L1 expression in CRC cells in vitro and in vivo (Figure 1) without altering its mRNA level (Figure S1, Supporting Information). In CRC patients with drinking history, PD‐L1 expression in their tumors was substantially higher than that of the non‐drinkers (Figure 1). Moreover, the abundance of CD3+ lymphocytes in tumor tissues was also significantly reduced in the drinkers than the non‐drinkers (Figure 1). Our results indicate that alcohol promotes CRC cells immune escape by inducing PD‐L1 expression. Herein, we suggest that CRC patients should give up drinking, and patients on PD‐1 immunotherapy are therefore advised to abstain from alcohol drinking to achieve the optimal anticancer effect.

ALDH2 is a major enzyme regulating alcohol metabolism and it is highly expressed in tumor tissues of patients consuming excessive alcohol.^[^ ^11^ ^]^ In our study, higher expression of ALDH2 was observed in tumor tissues from CRC patients with alcohol drinking history than the non‐drinkers (Table 1) and in mice with treated with alcohol (Figure 2). Importantly, we found alcohol promoted PD‐L1 expression was partly through ALDH2 (Figure 2). A strong and positive correlation between ALDH2 and PD‐L1 protein expression was also observed from the tumor specimens from CRC patients (Figure 2). From our cell line study, ectopic overexpression of ALDH2 expression was found to increase cell surface PD‐L1 protein expression (Figure S2, Supporting Information) without changing its mRNA level (Figure S1, Supporting Information). Previous studies revealed that ALDH2 plays the dual functions of promoting and inhibiting cancer. It can be antioncogenic by reducing the damaging effects of aldehydes, and promoting signaling cell survival especially in hepatocellular carcinoma.^[^ ^22^, ^23^ ^]^ However, some studies suggest that ALDH2 overexpression or high activity promotes cancer progression and MDR in clear‐cell renal cell carcinomas (ccRCC) and bladder cancer.^[^ ^24^, ^25^ ^]^ Our study also indicates that downregulation of ALDH2 was shown to increase tumor infiltration of CD3+ and CD8+ T lymphocytes and significantly suppress tumor growth and progression in our murine CRC model (Figure 2). Therefore, our results suggest that alcohol consumption induces ALDH2 expression in CRC to upregulate PD‐L1 and allow their escape from immune surveillance.

The regulatory mechanism between ALDH2 and PD‐L1 was further investigated. ALDH2 was shown to interact with the cytoplasmic region (amino acid residues 260–290) of PD‐L1 protein (Figure 3). We also found that the interaction between ALDH2 and PD‐L1 does not rely on the catalytic domain of ALDH2 (Figure 3). The critical structure domain of ALDH2 binding by PD‐L1 needs to be further research. Since ALDH2 increases PD‐L1 expression without affecting PD‐L1 mRNA level, ALDH2 is hypothesized to regulate PD‐L1 expression at the post‐transcriptional level. In this study, ALDH2 was found to increase the protein stability of PD‐L1 in CRC cell line (Figure 3). It has been reported that PD‐L1 protein is degraded via ubiquitin‐dependent and lysosome‐dependent mechanisms.^[^ ^26^, ^27^, ^28^ ^]^ Moreover, an E3 ubiquitin ligase SPOP has been recently demonstrated to mediate proteasome‐dependent degradation of PD‐L1 through binding to the motif region (283–290) of PD‐L1.^[^ ^14^ ^]^ Furthermore, either loss‐of‐function of SPOP or disruption of SPOP‐PD‐L1 interaction was found to promote upregulation of PD‐L1 in cancer cells and decrease lymphocytes infiltration.^[^ ^14^ ^]^ In another recent report, CKLF‐like MARVEL transmembrane domain containing protein 6 (CMTM6) was shown to interact with the transmembrane domain of PD‐L1 to prevent it from lysosome‐mediated degradation, thereby maintaining high expression of PD‐L1 at the plasma membrane.^[^ ^28^ ^]^ In our study, the proteasome inhibitor MG132 was shown to increase protein expression of PD‐L1 and ALDH2 whereas the lysosome inhibitor chloroquine had no effect (Figure 3). Moreover, our data also revealed that ALDH2 disrupted the poly‐ubiquitination of PD‐L1 (Figure 3). It was reported that SPOP mediated poly‐ubiquitination and degradation of PD‐L1 by binding to the T290 site within the cytoplasmic region of PD‐L1.^[^ ^14^ ^]^ By using a computational tool to predict possible amino acid residues for protein post‐translational modification, we identified K280 and K281 as novel ubiquitination sites on PD‐L1. Detailed biochemical investigation further confirmed that mutations at T290M or K280R+K281R significantly inhibited the ubiquitination and protein degradation of PD‐L1 (Figure 4). Furthermore, our data also showed that ALDH2 inhibited the ubiquitination modification of PD‐L1 at positions of K280, K281, and T290 (Figure 4). In addition, we demonstrated that ALDH2 inhibits the interaction of SPOP and PD‐L1, thereby preventing poly‐ubiquitination and enhancing protein stability of PD‐L1 (Figure 4).

In CRC murine model, alcohol‐induced mice responded well to PD‐1 blockade immunotherapy by increasing PD‐L1 overexpression (Figure 5). However, future works are warranted to confirm this observation and determine whether CRC patients with alcohol consumption would response to immune checkpoint inhibitors. Despite the remarkable clinical efficacy of immunotherapy achieved in a small subset of cancer patients, most CRC patients do not respond to PD‐1/PD‐L1 blockade immunotherapy. In order to expand the populations applicable to immunotherapy, it is necessary to develop novel treatment approaches to improve the efficacy of immune checkpoint inhibitors. Our results indicated that ALDH2 plays a critical role determining efficacy of PD‐1/PD‐L1 immune checkpoint blockade (Figure 5; Figure S4, Supporting Information). Inhibition of ALDH2 was found to reduce PD‐L1 expression but increase TILs infiltration (Figure 5; Figure S4, Supporting Information). This is the first study demonstrating the potentiation of PD‐1 blockade immunotherapy by combination of ALDH2 inhibition and PD‐1 blockade. According to the principle of reasonable drug application, two drugs would exhibit synergistic effect if they simultaneously inhibit same target by different pathway. ALDH2 may represent an attractive target for developing of novel cancer therapeutics combined with PD‐1/PD‐L1 blockades. In fact, the inhibition of ALDH2 was also shown to exhibit beneficial effect to chemotherapy in other cancer types. For instance, ALDH2 suppression in ccRCC with von Hippel–Lindau deficiency was shown to augment the effect of anthracycline chemotherapy.^[^ ^24^ ^]^ Inhibition of ALDH2 has also been shown to reverse drug resistance to cisplatin ^[^ ^29^ ^]^ and microtubule inhibitors.^[^ ^30^ ^]^ It is noteworthy that genetic polymorphisms in the ALDH2 gene contribute to alcohol‐induced facial flushing and susceptibility to alcoholism.^[^ ^31^ ^]^ In particular, the ALDH2*2 genotype is the most prevalent variant in East Asian descent.^[^ ^32^ ^]^ ALDH2*2 is known to reduce the aldehyde dehydrogenase enzymatic activity by ≈60–80% and low ALDH2 protein expression.^[^ ^33^, ^34^ ^]^ In addition, high TILs infiltration of tumor has been associated with favorable outcomes in CRC.^[^ ^35^ ^]^ Therefore, based on our finding about the low expression of ALDH2 relating to under expression of PD‐L1 and high TILs infiltration, genetic screening for ALDH2*2 may be adopted to identify sub‐population of CRC patients who are more likely to have favorable outcomes.

In summary, our work demonstrated that ALDH2 mediates alcohol‐induced CRC immune escape by preventing PD‐L1 from ubiquitin‐dependent degradation. Combination of ALDH2 inhibition and PD‐1 blockade, which increases TILs infiltration in tumor and prevents immune evasion, represents a novel strategy to potentiate immunotherapy in CRC patients with ALDH2 overexpression or heavy alcohol drinking history.

## 4 Experimental Section

##### Patient Samples

The use of human samples was approved by the Sun Yat‐sen University Cancer Center ethics committee (L102012019040H) and patients’ informed consent was obtained. Fresh CRC tissues were stored at −80 °C for western blot analysis. The pathological sections of CRC tissues were confirmed by pathologist and used for IHC detection by anti‐PD‐L1, anti‐CD3, anti‐CD8, and anti‐ALDH2 antibodies.

##### Cell Culture and Treatment

The human CRC cell lines, RKO, DLD1, HCT116, LOVO, HT29, SW480, SW620 and THC8307 were purchased from ATCC. All of them were cultured in RPMI‐1640 medium (Gibco) supplemented with 10% fetal bovine serum and 1% antibiotic mixture at 37 °C and 5% CO_2_.

##### Antibodies and Reagents

The primary antibodies used in western blot analysis included PD‐L1 anti‐human (#13684, CST), PD‐L1 anti‐mouse (17952‐1‐AP, Proteintech), ALDH2 (15310‐1‐AP, Proteintech), Myc tag (#2278, CST), Flag tag (20543‐1‐AP, Proteintech), HA tag (51064‐2‐AP, Proteintech), SPOP (16750‐1‐AP, Proteintech), and GAPDH (60004‐1‐Ig, Proteintech). Antibodies used in IHC assay were anti‐mouse CD3 (ab16669, Abcam), anti‐mouse CD8 (ab209775, Abcam), anti‐mouse granzyme B (ab4059, Abcam), anti‐human CD3 (Zhongshan Golden Bridge Biotechnology Co., Ltd., Beijing, China), and anti‐human CD8 (Zhongshan Golden Bridge Biotechnology Co., Ltd.). The secondary antibodies used in immunofluorescence assay were AF488‐anti‐rabbit (#8878, CST) and AF594‐anti‐mouse (Biotech). MG‐132 (M8699) and chloroquine (C6628) were purchased from Sigma. CHX (HY12320) was purchased from MedChem Express.

##### Plasmid Construction

The protocol of plasmid construction was described previously.^[32]^ Myc‐tagged ALDH2, Flag‐tagged ALDH2, Myc‐tagged SPOP, Flag‐tagged PD‐L1, and HA‐tagged ubiquitin constructs were cloned into pCMV vectors. The sgRNA oligonucleotides of ALDH2 were cloned into lentiviral vectors of lentiCRISPR v2. The shRNA oligonucleotides (Tables S1 and S2, Supporting Information) of ALDH2 were cloned into lentiviral vectors of pLKO.1. The ALDH2 was cloned into pCDH vectors. All plasmids were sequenced to confirm that the insert gene with the correct nucleotide sequence has been ligated at the desired base position within the vector backbone.

##### Cycloheximide Treatment

Cancer cells were incubated with CHX (20 µg mL^–1^) to block protein synthesis. Whole cell lysates were harvested from cell culture at the designated time points. PD‐L1 and ALDH2 protein stability were monitored by western blot analysis. The relative band intensity was quantified using ImageJ software.

##### Real‐Time RT‐PCR Analyses

Total RNA from cell lines was isolated using TRIzol reagent (Invitrogen). Quantitative RT‐PCR (RT‐qPCR) was performed using HiScript II Q Select RT SuperMix kit (Vazyme) for reverse transcription of total RNA and ChamQ SYBR qPCR Master Mix kit (Vazyme) for real‐time PCR. Real‐time PCR was performed with a LightCycler 480 Instrument (Roche). The primer sequences were listed as follows: ALDH2, forward 5′‐TCAAATTACAGGGTCAACTGCTA‐3′ and reverse 5′‐GCCCCCAACAGACCCCAATC‐3′; GAPDH, forward 5′‐GCATTGCCCTCAACGACCAC‐3′ and reverse 5′‐CCACCACCCTGTTGCTGTAG‐3′; PD‐L1, forward 5′‐TGGCATTTGCTGAACGCATTT‐3′ and reverse 5′‐TGCAGCCAGGTCTAATTGTTTT‐3′.

##### ALDH2 CRISPR sgRNA Sequences

sgRNA E2 F: CACCGCCAGTGGACGGATTGACGGT; sgRNA E2 R: AAACACCGTCAATCCGTCCACTGGC; sgRNA E5 F: CACCGCTGAAGAAGTCTCCGTCAAT; sgRNA E5 R: AAACATTGACGGAGACTTCTTCAGC.

##### Co‐Immunoprecipitation Assay

Cells were transfected with Flag‐PD‐L1, Flag‐ALDH2, Myc‐ALDH2, HA‐Ub, or Myc‐SPOP for 48 h and lysed with RIPA buffer (50 × 10^−3^ m Tris‐HCl (pH 8.0), 150 × 10^−3^ m NaCl, 1% NP‐40, 0.5% Na deoxycholate, 0.1% sodium dodecyl sulfate, supplemented with protease inhibitor cocktail). Protein concentration of cell lysate was measured by BCA Protein Assay Kit (Thermo Fisher Scientific) and 500 µg protein was used for incubation with either Anti‐FLAG Magnetic Beads (Sigma) or MYC antibody and magnetic beads (Roche) overnight at 4 °C. Co‐IP kit (Thermo Fisher Scientific, Pierce 26149) was used for detecting endogenous protein interaction. Next, samples were washed five times with RIPA buffer and treated with loading buffer for protein denaturation. Finally, samples were subjected to SDS–PAGE and western blot analysis.

##### Immunofluorescence Staining

Cells were cultured in confocal dishes for 48 h after transfection. Cells were fixed with 4% paraformaldehyde for 15 min at room temperature, washed twice with phosphate‐buffered saline (PBS), and permeabilized with 0.5% Triton X‐100 in PBS for 20 min at 4 °C. Afterwards, the dishes were incubated with the primary antibodies overnight at 4 °C after blocking with 5% bovine serum albumin for 1 h. Nonspecific antibody binding was removed by washing twice in PBS. This was followed by incubation with Alexa Flour 594‐labeled secondary antibody or PD‐L1 Alexa Flour 488 primary antibody for 1 h. Confocal images were generated on a ZEISS laser scanning confocal microscope (LSM880, Germany).

##### Immunohistochemistry

Fluorescent IHC staining of tissue samples was performed following standard protocol of deparaffinization, rehydration, antigen retrieval (ethylenediaminetetraacetic acid buffer, pH 9.0), permeabilization, blocking, antibody incubation, and color development. The immunohistochemical grading of ALDH2 and granzyme B was defined as –, +, ++, or +++ to represent negative, weak, intermediate, and strong staining, respectively, according to image intensity, and the density (0% = 0, 1–25% = 1, 26–50% = 2, and 51–75% = 3, >76% = 4) of positive cells. The immunoreactivity score was calculated as H‐score = (% of staining positive cells) (0–4) × (staining intensity) (0–3).^[^ ^36^ ^]^ An H‐score of (0–4) was defined as ALDH2 low, whereas an H‐score of (5–12) was defined as ALDH2 high. The percentage of PD‐L1‐positive tumor cells was detected as less than 1% and ≥1%, and the cutoffs of PD‐L1 were defined according to the clinical definition.^[^ ^37^ ^]^ The CD3‐ and CD8‐positive cells were quantified by the number of positive cells per field of vision (FOV, ×400).^[^ ^38^ ^]^

##### Flow Cytometry

Cells were harvested and washed twice in PBS and centrifuged at 1000 rpm for 5 min. The cells (1 × 10^6^) were incubated with antibodies including anti‐human PD‐L1 (329705, BioLegend, 1:50) conjugated with PE or anti‐mouse PD‐L1 (4347274, Invitrogen, 1:50) conjugated with PE or marched isotype IgG1 control at room temperature for 30 min. Data were analyzed by CytExpert software.

##### Enzyme‐Linked Immunosorbent Assay

Blood samples (1 mL) were collected using a serum separator tube from mice before euthanasia. The samples were allowed to clot at room temperature for 1 h before centrifugation for 10 min at 3000 × *g*. Serum was then transferred to a new tube and stored at –80 °C before analysis. IFN‐*γ* level in the serum samples was measured with a mouse IFN‐*γ* ELISA kit (MEIMIAN, China).

##### In Vivo Mice Tumor Models

The animal studies were approved by the Institutional Animal Care and Use Committee of Sun Yat‐sen University Cancer Center (YB2020‐009‐01). All animal studies were performed according to the guidelines provided by the Animal Ethics Committee of the Institute of Zoology, Chinese Academy of Sciences. For the first animal study evaluating the effect of alcohol consumption on PD‐1 blockade therapy, twenty four 4 week old BALB/c mice were randomly assigned into two groups: control group (intragastric administration of saline) and experimental group (intragastric administration of alcohol (5 g kg^–1^, once daily)).^[^ ^39^ ^]^ Two weeks later, mice were injected subcutaneously with mouse colon cancer CT26 cells (2 × 10^5^). When the tumor size reached ≈50 mm^3^, tumor‐bearing mice were randomly assigned into four groups: control group, alcohol‐induced group, PD‐1 antibody group, and alcohol+PD‐1 antibody group. PD‐1 antibody treatment was given 200 µg once every 3 days for a total of six times by intraperitoneal injection.

For the second animal study evaluating the effect of ALDH2 inhibition on PD‐1 blockade therapy, CT26 cells (3 × 10^5^) suspended in PBS were subcutaneously injected into the right flank of immunocompetent BALB/c (5–6 weeks old, female) mice or immunocompromised NSG mice (6 week old). When tumors reached ≈50 mm^3^, mice were randomized into different groups, involving control group, PD‐1 treatment group, si‐ALDH2 group, and si‐ALDH2+PD‐1 treatment group. The mice were treated with si‐ALDH2 (10 nmol) or its negative siRNA control (RiboBio) in 50 *μ*L saline buffer via direct injection into the tumor mass once every 3 days.^[^ ^40^ ^]^ The mice of PD‐1 treatment group were treated with anti‐PD‐1 antibody (BioXCell, 200 µg per mouse, intraperitoneally) every 3 days.^[^ ^41^ ^]^ Tumor size was measured with vernier caliper every other day and calculated by the formula: volume (mm^3^) = $\frac{1}{2}$(width)^2^ × length. The mice were sacrificed after the tumor size reached 2000 mm^3^ or the diameter of tumor ulcer exceeded 1 cm. Tumor tissues were removed from the body for IHC staining.

##### Statistical Analyses

Statistical analyses were performed using GraphPad Prism 8 (GraphPad Software) or the SPSS 22.0 software (IBM, USA). Data were presented as mean ± standard deviation (SD). For quantification of immune cell density, five fields of tumor sections with IHC staining were randomly selected and the positive cells were counted. Student's *t*‐test was used to compare the differences between the two groups. The Pearson's correlation between ALDH2 and PD‐L1 expression was calculated using GraphPad statistics software. Kaplan–Meier and log‐rank test were conducted to analyze the animal survival benefit. For western blot analysis, qRT‐PCR, ELISA, and other experiments, statistical testing was determined by the unpaired two‐tailed *t*‐test. Statistical information including *n* and *p* values is described in the text or the figure legends. All tests were considered statistically significant when ns = not significant, *p < 0.05, **p < 0.01, ***p < 0.001, and ****p < 0.0001.

## Conflict of Interest

The authors declare no conflict of interest.

## Author Contributions

H.Z., Y.X., and F.W. contributed equally to this work. H.Z. and L.F. contributed to the overall study design. H.Z., F.W., M.L., K.Y., and K.K.W.T. performed experiments in vitro. S.A. and S.W. performed animal treatments. C.Y., F.W., and M.X. performed the statistical analyses. M.C., D.C., and S.L. analyzed the clinical data and pathological sections. H.Z., K.K.W.T., and L.F. wrote the article. F.W. and Y.X. revised the article. All authors contributed to data interpretation and commented on the manuscript.

## Supporting information

Supporting Information

## Data Availability

The authenticity of this article was validated by uploading the key data onto the Research Data Deposit public platform (www.researchdata.org.cn), and the approval RDD number is RDDB2021001076.
