# Supplementary material for: Aldehyde Dehydrogenase 2 Mediates Alcohol‐Induced Colorectal Cancer Immune Escape through Stabilizing PD‐L1 Expression
Source: Adv Sci (Weinh). 2021 Mar 24;8(10):2003404. doi: 10.1002/advs.202003404 (PMC8132160; doi:10.1002/advs.202003404)
Supplement: Supplementary file 1 — Supporting Information [file ADVS-8-2003404-s001.pdf]

© 2021 Wiley-VCH GmbH

ADVANCED SCIENCE
Open Access

Supporting Information

for *Adv. Sci.*, DOI: 10.1002/advs.202003404

Aldehyde dehydrogenase 2 mediates alcohol-induced colorectal cancer immune escape through stabilizing PD-L1 expression

Hong Zhang[*], *Yuhui Xia*[*], *Fang Wang*[*], *Min Luo, Ke Yang, Shaobo Liang, Sainan An, Shaocong Wu, Chuan Yang, Da Chen, Meng Xu, Muyan Cai, Kenneth K W To, Liwu Fu*

Supplementary figure 1

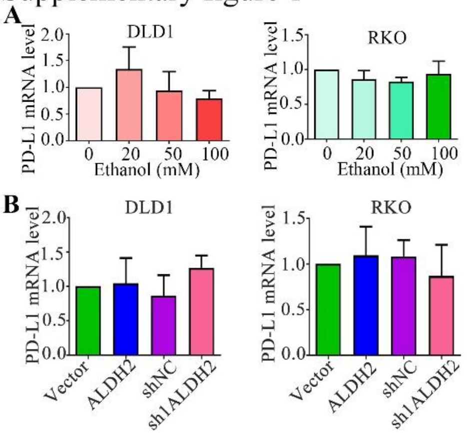

Supplementary figure 2

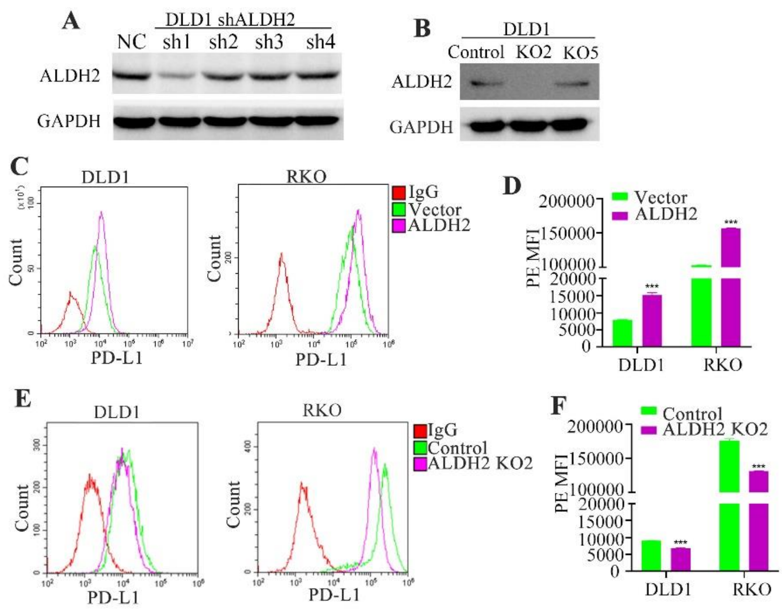

Supplementary figure 3

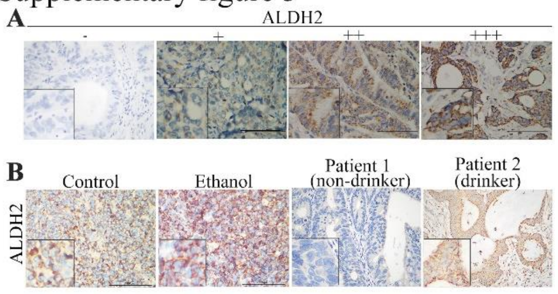

Supplementary figure 4

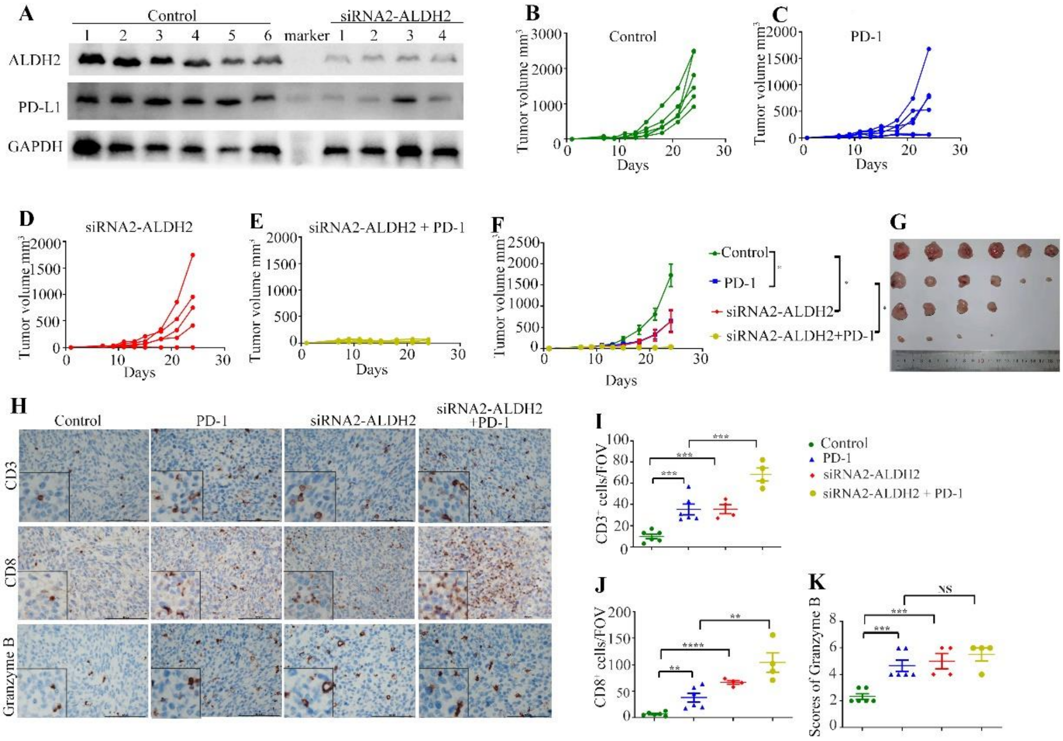

Supplementary Table 1

ALDH2 shRNA (human)	sequences
sh1 ALDH2 F:	5'-CCGGGGACATGGTCCTCAAATGTCTCTCG AGAGACATTTGAGGACCATGTCCTTTTTG-3'
sh1 ALDH2 R:	5'-AATTCAAAAAGGACATGGTCCTCAAATGT CTCTCGAGAGACATTTGAGGACCATGTCC-3'
sh2 ALDH2 F:	5'-CCGGGCAGATCATTCCGTGGAATTTCTCG AGAAATTCCACGGAATGATCTGCTTTTTG-3'
sh2 ALDH2 R:	5'-AAttCAAAAAGCAGATCATTCCGTGGAATT TCTCGAGAAATTCCACGGAATGATCTGC-3'
sh3 ALDH2 F:	5'-CCGGGAAACGTGGTTGTGATGAAGGCTC GAGCCTTCATCACAACCACGTTTCTTTTTG-3'
sh3 ALDH2 R:	5'- AATTCAAAAAGAAACGTGGTTGTGATGAA GGCTCGAGCCTTCATCACAACCACGTTTC-3'
sh4 ALDH2 F:	5'-CCGGGCAGATCATTCCGTGGAATTTCTCG AGA-3'
sh4 ALDH2 R:	5'-AATTCCACGGAATGATCTGCTTTTTG-3'

Supplementary Table 2

ALDH2 shRNA (mouse)	sequences
sh1 ALDH2 F:	5’-CCGGCCTGAAATGTCTCCGCTATTACTCG AGTAATAGCGGAGACATTTCAGGTTTTTG-3'
sh1 ALDH2 R:	5'-AATTCAAAAACCTGAAATGTCTCCGCTAT TACTCGAGTAATAGCGGAGACATTTCAGG-3'
sh2 ALDH2 F:	5'-CCGGCGGGCCAATGATTCTAAGTATCTCG AGATACTTAGAATCATTGGCCCGTTTTTG-3'
sh2 ALDH2 R:	5'-AATTCAAAAACGGGCCAATGATTCTAAGT ATCTCGAGATACTTAGAATCATTGGCCCG-3'
sh3 ALDH2 F:	5'-CCGGCCAGTGATGCAAATCCTCAAACTCG AGTTTGAGGATTTGCATCACTGGTTTTTG-3'
sh3 ALDH2 R:	5'-AATTCAAAAACCAGTGATGCAAATCCTCA AACTCGAGTTTGAGGATTTGCATCACTGG-3'

Supplementary Figure 1. PD-L1 mRNA expression. **A,** qPCR validation of PD-L1 expression after treatment with alcohol at different concentrations for 48 h. **B**, Expression of PD-L1 mRNA in vector, ALDH2, shNC, and sh1 ALDH2-transfected DLD1 and RKO cells.

Supplementary Figure 2. ALDH2 upregulated PD-L1 protein expression on cell membrane surface. **A,** Western blot analysis showing the down-expression of ALDH2 by 4 different shRNA in DLD1 cells. **B**, Western blot analysis the effective knockout of ALDH2 by two sgRNA. **C, E**, Flow cytometry assay showing the cell membrane surface expression of PD-L1 after ectopic ALDH2 overexpression or deletion in DLD1 and RKO cells. **D**, **F**, Histograms showing the mean fluorescence intensity value (MFI) $\pm$ SD values from three biological replicates in the flow cytometry assay. (Student's *t* test, **P* < 0.05; ***P* < 0.01; ****P* < 0.001.)

Supplementary Figure 3. IHC staining images showing the different ALDH2 protein expression in tumor tissues obtained from CRC patients. **A,** Rating of IHC intensity staining comparing relative ALDH2 expression (-, +, ++, +++ representing negative, weak, intermediate and strong staining intensity, respectively). **B**, The expression of ALDH2 in tumor tissues of CRC patients with or without alcohol drinking history.

Supplementary Figure 4. Another siRNA of ALDH2 enhanced efficacy of PD-1 blockade in CT26 tumor model. **A,** Western blot analysis showing ALDH2 and PD-L1 protein expressions in tumor tissues from control and siRNA2-ALDH2 treated mice. **B-E**, Tumor volume of mice treated with control (n = 6), anti-PD-1 mAb (n = 6), siRNA2-ALDH2 (n = 6) and siRNA2-ALDH2 combined anti-PD-1 mAb therapy (n = 6) as indicated were measured every two days and plotted individually. **F**, The tumor volume curves of four different groups. **G**, Photo showing tumors excised from different treatment groups at termination of study. H, Representative IHC staining results of CD3, CD8, and granzyme B from four treatment groups as indicated. **I**, **J** The number of CD3 and CD8 positive cells per field of vision (FOV) in tumor tissues from IHC analysis (400×). **K**, The immunoreactivity score of granzyme B in tumor tissues of different treatment groups (400×). Error bars represent SD. (Student's *t* test, **P* < 0.05; ***P* < 0.01; ****P* < 0.001; *****P* <

0.0001.)
